# Inferring Intra-Community Microbial Interaction Patterns from Metagenomic Datasets Using Associative Rule Mining Techniques

**DOI:** 10.1371/journal.pone.0154493

**Published:** 2016-04-28

**Authors:** Disha Tandon, Mohammed Monzoorul Haque, Sharmila S. Mande

**Affiliations:** Bio-Sciences R&D Division, TCS Research, Tata Consultancy Services Limited, 54-B, Hadapsar Industrial Estate, Pune 411013, Maharashtra, India; University of Hyderabad, INDIA

## Abstract

The nature of inter-microbial metabolic interactions defines the stability of microbial communities residing in any ecological niche. Deciphering these interaction patterns is crucial for understanding the mode/mechanism(s) through which an individual microbial community transitions from one state to another (e.g. from a healthy to a diseased state). Statistical correlation techniques have been traditionally employed for mining microbial interaction patterns from taxonomic abundance data corresponding to a given microbial community. In spite of their efficiency, these correlation techniques can capture only 'pair-wise interactions'. Moreover, their emphasis on statistical significance can potentially result in missing out on several interactions that are relevant from a biological standpoint. This study explores the applicability of one of the earliest association rule mining algorithm i.e. the 'Apriori algorithm' for deriving 'microbial association rules' from the taxonomic profile of given microbial community. The classical Apriori approach derives association rules by analysing patterns of co-occurrence/co-exclusion between various '(subsets of) features/items' across various samples. Using real-world microbiome data, the efficiency/utility of this rule mining approach in deciphering multiple (biologically meaningful) association patterns between 'subsets/subgroups' of microbes (constituting microbiome samples) is demonstrated. As an example, association rules derived from publicly available gut microbiome datasets indicate an association between a group of microbes (Faecalibacterium, Dorea, and Blautia) that are known to have mutualistic metabolic associations among themselves. Application of the rule mining approach on gut microbiomes (sourced from the Human Microbiome Project) further indicated similar microbial association patterns in gut microbiomes irrespective of the gender of the subjects. A Linux implementation of the Association Rule Mining (ARM) software (customised for deriving 'microbial association rules' from microbiome data) is freely available for download from the following link: http://metagenomics.atc.tcs.com/arm.

## Background

Recent advances in high-throughput sequencing technologies have enabled life sciences researchers to investigate structure and functional relationships between various organisms constituting a microbial ecosystem (referred to as microbiome). Deciphering such relationships and interpreting them in the context of the studied environment is a prime objective of contemporary microbiome research initiatives. Inferences drawn by analysing (and comparing) patterns of co-occurrence of various microbes (taxa) constituting microbial communities (sampled across space and/or time) are expected to aid in (1) obtaining better understanding of inter-microbial interactions in various environmental niches (2) identifying specific microbial interaction patterns that directly or indirectly determine the stability of a given microbial community, and (3) studying and understanding the mode/mechanism(s) by which a microbial community transitions from one state to another (e.g. from healthy to a diseased state).

Typically, identification of microbial interactions (i.e. their co-occurrence patterns) is done by employing correlation coefficient measures such as Pearson, Kendall, Spearman, Kullback-Leibler Distance, or a dissimilarity measure like Bray Curtis. To eliminate spurious correlation artefacts that may result due to differences in sequencing and/or sampling depth, the input (taxa) abundance matrix is usually subjected to various normalization techniques prior to computing correlation coefficients. Rows (in the abundance matrix) corresponding to organisms having (a) null observations in a majority of samples, or (b) low abundance values (below a specified threshold), are usually purged from the abundance matrix prior to analysis.

A wide variety of algorithms and statistical tools are currently available for visualizing and analysing networks that are generated using results obtained in such correlation analyses. These tools help in identifying pairs of microbes having positive/negative correlations in their abundance patterns and visualize the identified pairs in form of interaction networks. Nodes and edges in such networks correspond to pairs of microbes having a positive/negative correlation. Recent methods like CoNet [1] improve the confidence of the predicted interactions by employing an ensemble of correlation and similarity measures that are integrated along with suitable randomisation and test correction routines (e.g. Simes method, FDR correction, etc.)

The overall topology and patterns of inter-connectivity observed in the generated network(s) provide valuable biological insights with respect to 'pair-wise' microbial interactions. Although, such insights help in predicting/understanding the functional/metabolic exchanges that potentially occur between the identified member pairs, in a real-world scenario, the nature/complexity of inter-microbial interactions is significantly beyond simple 'pair-wise' interactions. It is well known that the metabolic potential of any naturally occurring microbial community is defined by the combined action of several 'groups/subsets' of resident microbes that interact due to mutual dependencies in terms of shared reactant/intermediate metabolites and other essential enzymes. This scenario calls for exploration, development and application of novel algorithms, tools, and suitable data-mining techniques that can go beyond prediction and analysis of simple 'pair-wise' interactions.

Given the complexity of interactions within microbial communities, the aim of the present study is to explore the applicability of one of the earliest association rule mining algorithm, the 'Apriori algorithm' (R. Agrawal and R. Srikant, 1994), for deriving 'association rules' from microbial abundance data. The derived rules are expected to indicate biologically meaningful co-occurrence/co-exclusion patterns between 'subsets/subgroups' of microbes/ taxa constituting these microbiome samples. Various customisations performed for adapting the Apriori approach for microbiome data are appropriately described. Validation experiments that highlight the efficiency of this 'customised' Apriori approach in deciphering association rules between various microbial groups in real-world microbiome datasets are also detailed.

## Methods

The principle and methodology of the classical Apriori approach is first summarised using the classical 'market-basket' analysis problem as a representative example. An introduction to various parameters/thresholds (employed in the Apriori approach) that determine the overall confidence of the derived/mined association rules is also included in this summary. Subsequently, an explanation of various customisations that were done for making the Apriori rule mining approach efficient and amenable for analysing microbial abundance data is provided.

### Principle and methodology of the classical Apriori approach

The classical Apriori algorithm is based on the principle of frequent pattern mining. It is primarily a deductive approach that derives/extracts conclusions (or 'rules') by identifying the presence of correlations, frequent patterns, and/or associations between various (subsets of) features/items in existing information. Performing Apriori analysis on given information/data involves the following two steps -

1. **Candidate-set generation**. This step involves finding those features/items that occur (in the given information/data) with a frequency that exceeds a specified threshold (referred to by the term 'support-count'). Occurrence of a feature is defined in terms of its presence/absence in the given data. Such a group of frequently occurring feature/items constitute the 'candidate-set'. In the classical 'market basket' example, the Apriori property is employed with the objective of analysing the purchasing behaviour of customers by observing their individual co-purchasing patterns with respect to certain items (or) groups of items. For this purpose, data pertaining to purchase history (of various customers) is first analysed for generating a candidate-set that contains items that are 'frequently' purchased by customers (the frequency being defined by the 'support-count' parameter)
2. **Associative Rule mining**. This steps analyses items in the candidate set for identifying/mining 'association' rules which essentially indicate the presence of a group given the presence of the other. This mining process involves the following steps. Initially, all possible groups/subsets that can potentially be formed using items in the candidate-set are first generated. Conditional probabilities between each pair of 'groups' are subsequently computed. Rules are generated based on pairs whose conditional probability value exceeds a user-defined threshold (the probability threshold being defined by a parameter referred to as 'confidence value'). In the context of the market-basket example, co-purchasing patterns between various items in the candidate item set are identified/ mined. The association rules mined in this example indicate the likely-hood of a customer purchasing a group of items given that he/she has purchased another item or group of items.

### Adapting Apriori approach for mining association rules from microbiome data

The classical 'association rule mining' process was customised for identifying analogous associations between microbial groups (in microbiome samples) in the following manner.

#### Customisation of candidate set generation process

**Fig 1:**
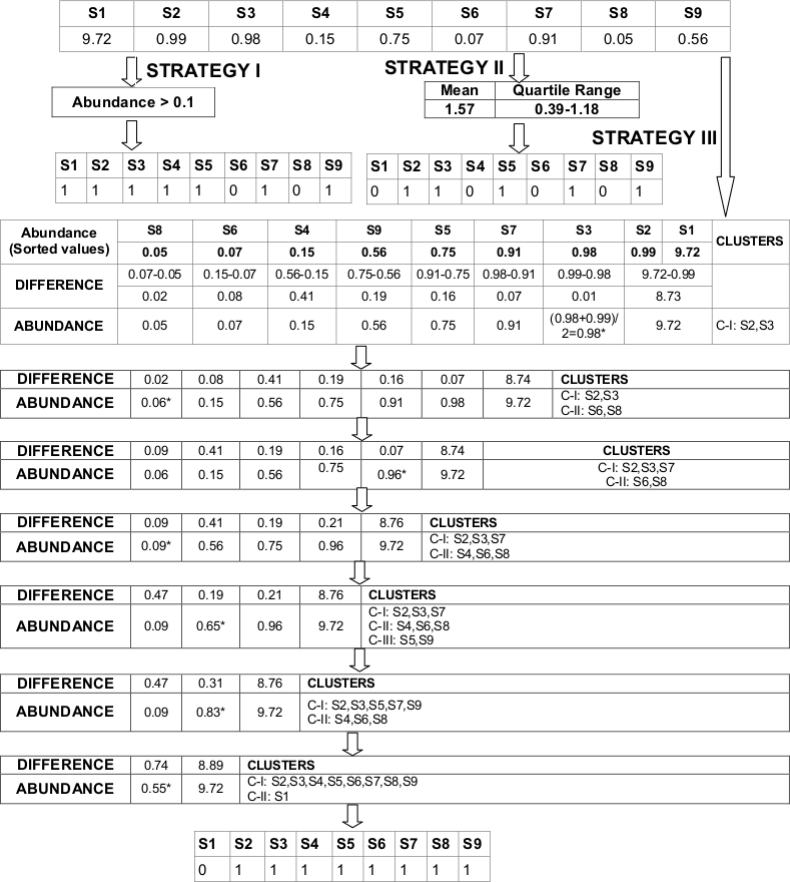
Schematic diagram depicting the three strategies employed for indicating the presence/ absence of a taxon. Schematic diagram depicting the three strategies employed for indicating the presence/ absence of a taxon (in various samples) based on their abundance values (in the respective samples). The first strategy (depicted in section A), relies only on the abundance proportion of the taxa in each sample. A taxon whose (normalized) abundance proportion (in a sample) exceeds 0.1% is considered as 'present' (in that sample). In the second strategy (depicted in section B), a taxon is reported as 'present' (in a sample) only if its abundance value (in that sample) lies between the 2nd and 3rd quartile range of the computed mean/median value. Strategy 3 (depicted in section C) involves computing Manhattan distances between individual abundance values of a taxon (in each of the samples) and then hierarchically clustering the samples on basis of the computed distances. Given that hierarchically clustering in this case involves only singular abundance values, the clustering can be achieved by progressively merging sample pairs with the least distance. The sorting mechanism indicated in the figure helps in making the distance calculation process less time consuming (i.e. computationally efficient). Note that the final two clusters obtained indicate that the taxon is reported as 'present' in all samples except for Sample S1.

Microbial abundance data does not capture the abundances of various microbes/taxa (constituting an environmental sample) in terms of 'mere' presence/absence. Appropriate methods are therefore required to decide a suitable (minimum) abundance threshold for reporting a taxon to be 'present' in the sample being analysed. For individual taxa, the (minimum) abundance threshold was computed/ defined using one of the following parameters/strategies. These strategies are illustrated in Fig 1 –

1. Strategy I: A taxon whose (normalized) abundance proportion (in a sample) exceeds 0.1% is considered as 'present' (in that sample).
2. Strategy II: For a taxon, compute its mean/median abundance value across various samples. A taxon is reported as 'present' (in a sample) only if its abundance value (in that sample) lies between the 2^nd^ and 3^rd^ quartile range of the computed mean/median value.
3. Strategy III: A distance matrix is created based on Manhattan distances computed between individual abundance values of a taxon (in each of the samples). The distance values are then hierarchically clustered (and progressively merged) until 2 clusters remain. The taxon is reported as 'present' only for those samples whose abundance values constituted the biggest cluster. In case of a tie, the hierarchical clustering (and progressive merger) process is continued until a stage wherein the final resulting clusters differ in size.

The support-count parameter (similar to that used in the classical Apriori procedure), is subsequently employed to retain only those taxa that are reported as 'present' in at least 65% (i.e. close to two-thirds) of the samples constituting a given microbiome dataset. Taxa retained in this manner constitute the final 'candidate (taxa) set'.

#### Customisation of rule mining procedure

**Fig 2:**
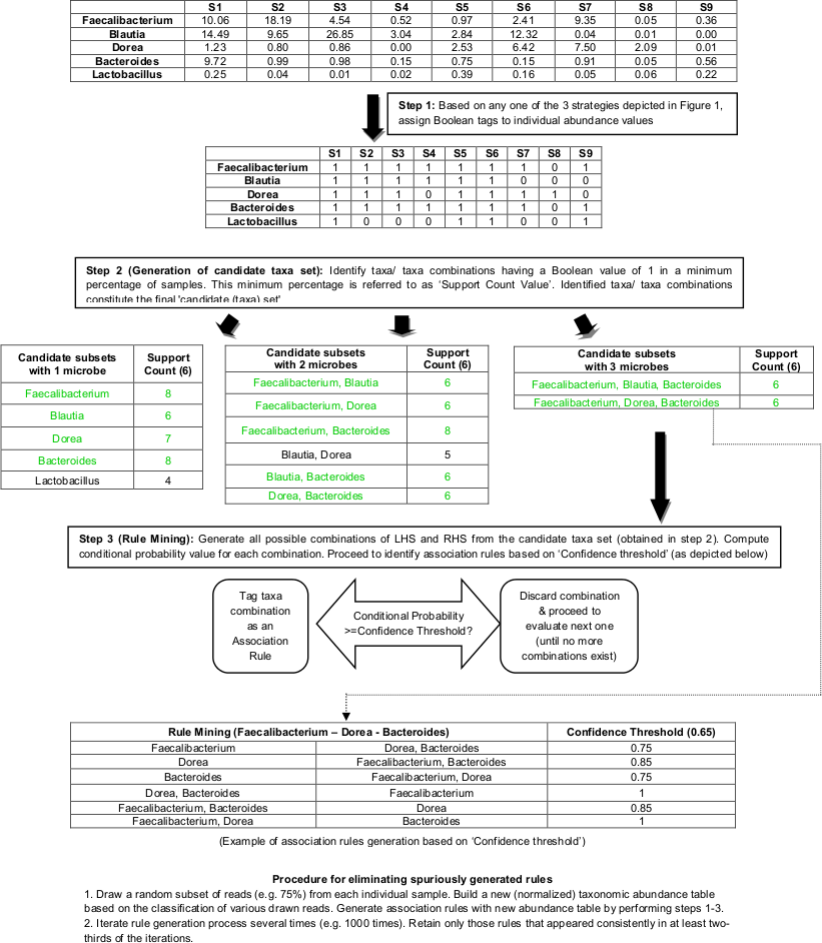
Schematic work-flow depicting the associative rule mining procedure customised for microbial abundance data. A schematic work-flow depicting the associative rule mining procedure that has been customised for microbial abundance data. The work-flow has been explained using an initial example abundance matrix which depicts normalized proportions of five distinct microbes in nine microbiome samples (S1 to S9). The subsequently indicated Boolean matrix (wherein taxa abundances have been indicated by presence/absence values i.e. 0 and 1) was generated by employing strategy I in which taxa whose normalized abundance were greater than 0.1 are considered as 'present'. The subsequent steps represent the process of candidate set generation. The depicted example indicates the use of a Support Count Value of 6. Taxa whose Support Count Value exceeded 6 (indicated in green font) eventually constitute the candidate set. The final matrix represents the sole association rule generated after validating various taxa combinations (in the candidate set) for confidence value threshold. Note that this rule is generated only if all possible (indicated) taxa combinations exceed the confidence value threshold.

In spite of retaining the classical rule mining procedure using a confidence value of 0.65, an additional ‘scoring process’ was adopted for filtering out spuriously predicted rules. This scoring process involved the following steps. A predetermined proportion of reads (e.g. 75%) were drawn from each individual sample (from amongst multiple samples constituting a microbiome dataset) and a normalized taxonomic abundance table was generated based on the classification of various drawn reads. Rules were generated from this abundance table. This process of rule generation was repeated several times (the number of repetitions defined by the end-user). Rules that appeared consistently in at least two-thirds of the iterations are retained. Fig 2 schematically depicts with an example, the associative rule mining process that has been customised for microbial abundance data.

**Fig 3:**
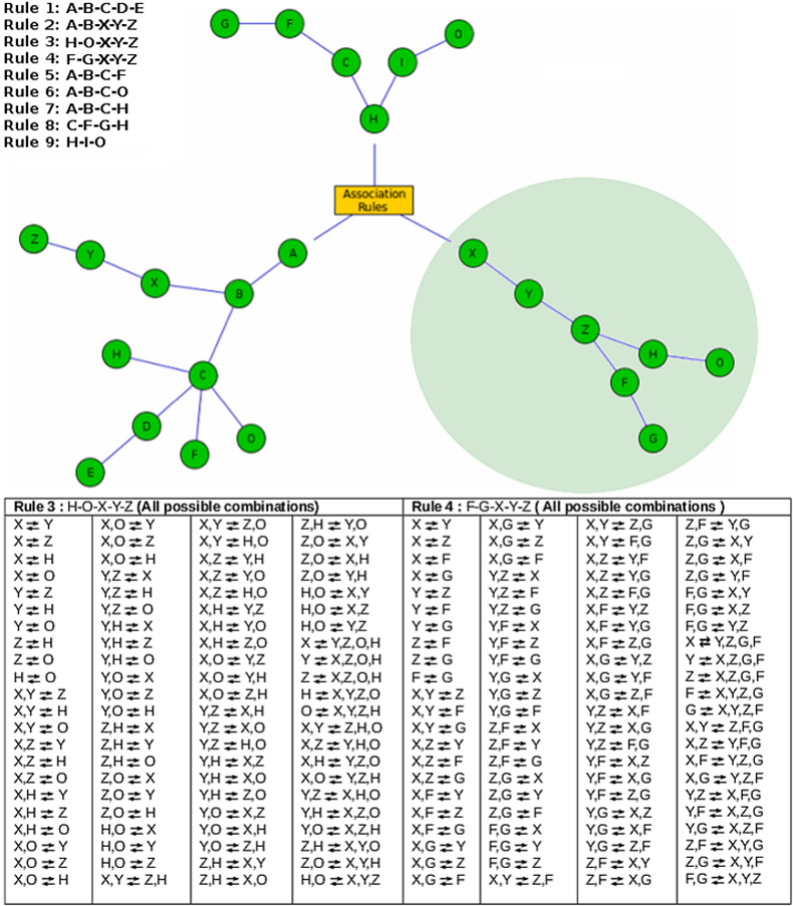
Minimalist graphical representation of associative rules involving 3 or more genera. A 'minimalist' graphical representation of associative rules (involving 3 or more genera) generated from an example dataset containing 26 genera named alphabetically (A to Z). Rules indicated in this example involve only 13 out of 26 genera. It is pertinent to note here that genera (and/ or groups of genera) constituting an individual rule share an all-to-all associative relationship. For examples rule 3 (involving 5 genera viz. X, Y, Z, H, and O) not only indicates an associative relationship between all possible genera pairs, but also between all possible combinations of genera. For the purpose of clarity, an exhaustive list of such combinations (possible from rule 3) is provided in the table depicted in Fig 3. As indicated, rule 3 (for instance) indicates an association between the abundances of genera pair (X, Y) and the genera group (Z, H, and O). Given that Fig 3 illustrates a 'minimalist' graphical representation of all associative rules, genera X, Y, and Z (common to rules 3 and 4) are shown only once in the circled portion of the illustrated figure. The table depicted in Fig 3 also provides an exhaustive list of taxa and combinations of taxa generated from rule 4.

Fig 3 provides a 'minimalist' graphical representation of associative rules (involving 3 or more genera) generated from an example dataset containing 26 genera named alphabetically (A to Z). This minimalist representation, analogous to a ‘concept linkage’ diagram which represents connections between co-occurring words/topics/concepts identified through a text-mining exercise, allows users to easily visualise/interpret the co-occurrence and potential interactions between ‘associated’ genera. Rules indicated in this example involve only 13 out of 26 genera. It is pertinent to note here that genera (and/or groups of genera) constituting an individual rule share an all-to-all associative relationship. For examples rule 3 (involving 5 genera viz. X, Y, Z, H, and O) not only indicates an associative relationship between all possible genera pairs, but also between all possible combinations of genera. For the purpose of clarity, an exhaustive list of such combinations (possible from rule 3) is provided in section A of the table depicted in Fig 3. As indicated, rule 3 (for instance) indicates an association between the abundances of genera pair (X, Y) and the genera group (Z, H, and O). Given that Fig 3 illustrates a 'minimalist' graphical representation of all associative rules, genera X, Y, and Z (common to rules 3 and 4) are shown only once in the circled portion of the illustrated figure. Section B of the table depicted in Fig 3 provides an exhaustive list of taxa/taxa group combinations generated from rule 4.

## Results

The Apriori algorithm, customised for deciphering association rules from microbiome abundance data, was evaluated using the following datasets -

### A. Prebiotic datasets

445 samples from publicly available microbiome datasets from two previous studies [2, 3] which had analysed the impact of prebiotics on the gut microbiome. In both studies, samples were segregated into three groups (pre, during, and post). While the first group comprised gut microbiome (i.e. stool) samples taken from subjects prior to the administration of specific prebiotic supplements, the second and third groups had samples obtained during the administration and post-administration phase respectively.

### B. HMP datasets

Gut Microbiome datasets from the HMP i.e. the Human Microbiome Project [4]. Available datasets (containing a total of 309 samples), based on subject metadata, were divided into two groups viz. Females and Males.

The rationale behind choosing the above mentioned datasets for evaluating the customised Apriori algorithm is the following -

1. All evaluation datasets correspond to microbiome samples taken from the human gut. At the current juncture, there is availability of several research papers and reviews that provide information with respect to the physiological interdependence of various bacterial genera residing in the human gut. Such information would primarily help in validating whether the association rules generated contain the expected set of interacting genera.
2. In prebiotic datasets, comparison of the association rules generated at various phases (i.e. pre, during, and post administration) would help in identifying a core set of genera that are always seen to be associated irrespective of the transition state. Moreover, the generated association rules would help in understanding microbial interaction dynamics associated with this transition.
3. HMP datasets were obtained from subjects belonging to the same geography. A comparison of association rules generated using datasets from male and female subjects would help in observing patterns of similarities/differences between human gut microbial interactions in male and female subjects.

Taxonomic assignments for individual reads (in all samples) were obtained using RDP classifier (version 2.10; bootstrap confidence threshold: 0.8). Abundance tables were generated based on the number of reads assigned to individual genera in each of the samples. S1 File provides all abundance tables (used in the present study) in the form of a zip archive. Generated tables (corresponding to individual datasets) were provided as input to the customised Apriori implementation. During the candidate taxa generation process, strategy 1 was adopted for defining the (minimum) abundance threshold for individual taxa. Support count value of 65, and a rule-mining confidence value of 0.65 were used as parameters during the rule-mining process.

**Fig 4:**
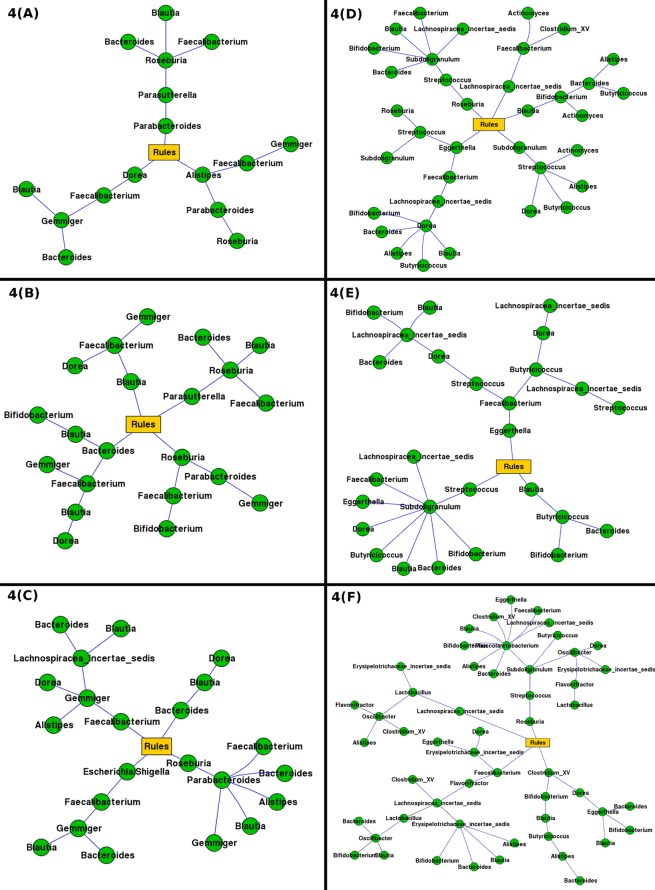
Associative rules (involving 3 or more genera) generated from the prebiotic datasets. A graphic representation of associative rules (involving 3 or more genera) generated from the prebiotic datasets. Parts A, B and C depict association rules generated from the Chinese prebiotic datasets [2]. Parts D, E and F depict association rules generated from the Japanese prebiotic datasets [3].

**Fig 5:**
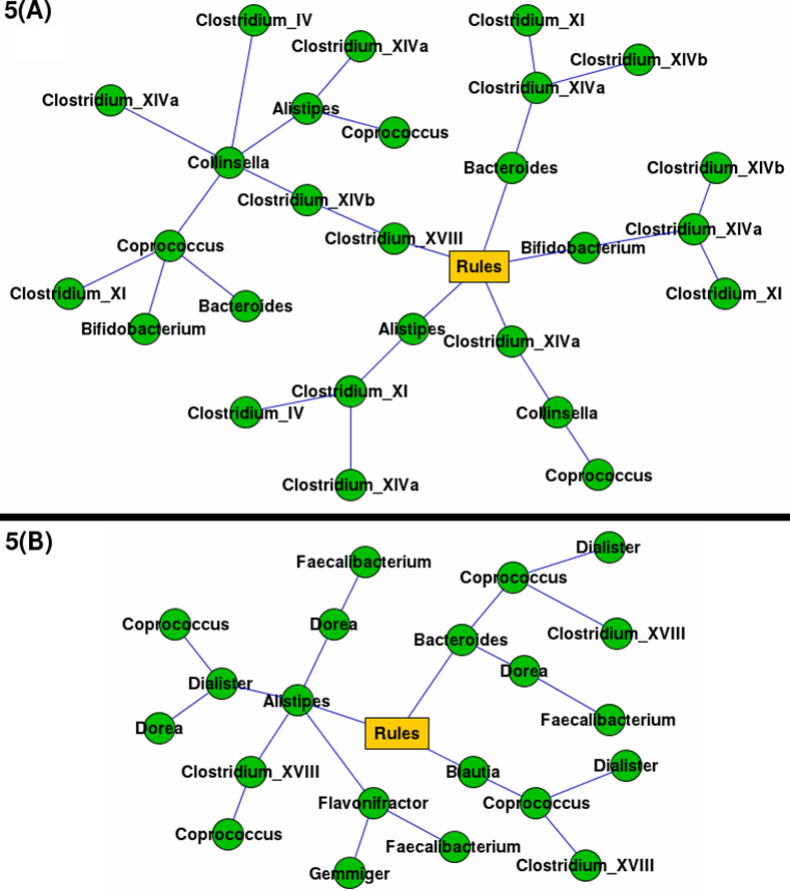
Associative rules (involving 3 or more genera) generated from the HMP datasets. A graphic representation of associative rules (involving 3 or more genera) generated from the HMP datasets [4]. Parts A and B depict association rules generated from samples corresponding to male and female subjects respectively.

For each of the validation datasets, Table 1 summarizes information pertaining to (a) the number of samples, (b) the number of generated association rules (total as well as rules that involve 3 or more genera), (c) the unique number of microbial genera involved in the identified association rules, and (d) execution time. Figs 3–5 provide a graphic representation of associative rules (involving 3 or more genera) generated from individual validation datasets used in this study.

**Table 1:** Number of association rules generated using the Apriori rule mining approach with various datasets. Summarised information pertaining to (a) the number of samples, (b) the number of generated association rules (total as well as rules that involve 3 or more genera), (c) the unique number of microbial genera involved in the identified association rules, (d) execution time, and (e) the number of rules generated using an alternative rule mining strategy (detailed in discussion section of the manuscript).

**Study**		Number of Samples	Number of Taxa	Execution Time for ARM (minutes)	Number of Rules with ARM		Number of rules with the alternative strategy (3 or more microbes)
					Total number of rules	Rules with 3 or more microbes	
**Prebiotics Chinese**	**Pre administration**	94	24	19	52	15	1
	**During administration**	97	23	17	43	11	1
	**Post administration**	93	25	21	56	14	0
**Probiotics Japanese**	**Pre administration**	19	22	5m 23s	36	5	0
	**During administration**	72	27	1m 14s	64	13	1
	**Post administration**	70	24	6m 14s	66	12	0
**Human Microbiome Project (HMP)**	**Female**	126	26	1m 17s	71	11	3
	**Male**	183	32	1m 37s	76	14	4

### Results obtained with Prebiotic datasets

With respect to the number of rules having three or more genera, graphs generated from both studies (depicted in Fig 4) primarily indicate associations between groups of genera that share related physiological functions. For e.g. in both studies related to prebiotics, in datasets that were obtained prior to the administration of the prebiotic, the generated rules indicate an association between the genera Blautia, Faecalibacterium, and Dorea. These three genera are physiologically associated in the following manner. Blautia is known to produce acetate from hydrogen and carbon dioxide [5]. Acetate, in turn, is utilized as an energy source by Faecalibacterium which results in generation of butyrate as an end-product [6]. Butyrate is known to induce mucin synthesis [7]. Given that mucosal layer thickness is defined by a fine balance between mucin synthesis and degradation rates, it is interesting to find an association between Faecalibacterium and Dorea, a genus known for its mucin degrading capabilities [8].

Results from datasets that were obtained during the administration of the prebiotic supplement indicate similar types of associations described above. One distinct change is the inclusion of Bifidobacterium genera in the association rules generated from the Chinese pre-biotic [2] datasets. The likely role of this genus is to regulate the levels of glucosidases [9]. These enzymes, typically produced during metabolism of fructo-oligosaccharides (i.e. prebiotic) by acetate producing microbes (e.g. Bacteriodes, Blautia etc.), unmask mucin-associated carbohydrate receptors. Such an action increases bacterial adherence rates which in turn lead to a state of infection. Interestingly, graphs corresponding to the post-administration phase (of both studies) indicate an increase in the number of associations between the same set of bacteria that were present before or during the administration phase.

In summary, results discussed above indicate that the customised Apriori approach was able to generate association rules containing groups of genera that are known to have mutualistic metabolic associations among themselves. Furthermore, the rules indicate that 'Faecalibacterium-Dorea-Blautia' group always share an associative relationship more or less irrespective of the transition state indicating thereby reflecting core functional dependencies. It will be interesting to see if the same functional interdependence is observed in gut microbiomes taken from diseased states (from subjects in the same geographies).

### Results obtained with HMP datasets

As seen in the results obtained in the prebiotic datasets, graphs generated from HMP studies (Fig 5A and 5B) also indicate rules that capture relationships between a set of bacteria that have well-known synergistic associations. There are instances of identical/similar rules generated in both datasets indicating 'conserved' association patterns between microbial community members in gut samples from amongst subjects originating from the same geography (irrespective of their gender). For example, genera sharing functions involving acetate and butyrate production/degradation, viz. Bacteriodes, Clostridium XVIII, and Coprococcus, are again observed to be associated [6, 10].

In order to evaluate if the same set and similar number of rules are generated at higher threshold values, the above experiments (on prebiotic as well as HMP datasets) were also performed with higher ‘confidence value’ and ‘support count’ thresholds. Tables 2 and 3 depict the number of rules generated as a function of increasing threshold values. Overall, as expected, results in this table indicate higher threshold values result in fewer rules being generated. However, in spite of the randomisation procedure adopted during the final rule scoring step, higher thresholds did not result in generating newer rules. This indirectly reflects the robustness/ utility of the final scoring process in removing false positive predictions. Assuming that values of 65 (for support count) and 0.65 (as probability threshold) provide acceptable /reasonable confidence (with respect to the generated rules), the current experiments used these values as thresholds.

**Table 2:** Number of association rules generated from the prebiotics dataset with various run-time thresholds. Number of association rules generated using the Apriori rule mining approach on the prebiotics dataset at various values of support count and confidence thresholds. Table also depicts variations in number of rules due to adoption of various strategies that define the minimum abundance threshold for individual taxa to be considered for rule mining.

Strategy	Support Count	Confidence threshold			
		0.65	0.70	0.75	0.80
**Strategy I**	**65**	7	7	6	7
	**70**	3	3	3	3
	**75**	3	4	3	3
	**80**	4	2	2	1
**Strategy II**	**65**	6	5	5	5
	**70**	3	3	2	2
	**75**	2	2	2	1
	**80**	1	1	1	1
**Strategy III**	**65**	0	0	0	0
	**70**	0	0	0	0
	**75**	0	0	0	0
	**80**	0	0	0	0

**Table 3:** Number of association rules generated from the HMP (male) dataset with various run-time thresholds. Number of association rules generated using the Apriori rule mining approach on the HMP (male) dataset at various values of support count and confidence thresholds. Table also depicts variations in number of rules due to adoption of various strategies that define the minimum abundance threshold for individual taxa to be considered for rule mining.

Strategy	Support Count	Confidence threshold			
		0.65	0.70	0.75	0.80
**Strategy I**	**65**	86	85	85	84
	**70**	60	60	60	60
	**75**	12	12	11	11
	**80**	6	5	5	5
**Strategy II**	**65**	78	77	76	64
	**70**	52	50	50	50
	**75**	12	10	9	9
	**80**	5	3	3	3
**Strategy III**	**65**	7	7	6	6
	**70**	6	6	5	4
	**75**	4	4	3	3
	**80**	86	85	85	84

On a similar note, the process of candidate taxa generation in the above experiments utilized strategy I (Fig 1) for defining the minimum abundance threshold for individual taxa. Tables 2–4 provide a summary of results indicating the number of rules generated upon employing the other two strategies (Fig 1) on the complete HMP dataset. The values in this table also indicate the number of predicted rules (and the time required to generate the same) as a function of change in the number of iterations during the final scoring process. Results indicate the following trends–

**Table 4:** Number of association rules generated from the HMP (full) dataset with various run-time thresholds. Number of association rules generated using the Apriori rule mining approach on the HMP (full) dataset at various values of support count and confidence thresholds. Table also depicts variations in number of rules due to adoption of various strategies that define the minimum abundance threshold for individual taxa to be considered for rule mining.

Strategy	Support Count	Confidence threshold			
		0.65	0.70	0.75	0.80
**Strategy I**	**65**	15	14	14	14
	**70**	13	12	12	11
	**75**	10	9	9	8
	**80**	5	4	3	3
**Strategy II**	**65**	12	12	12	12
	**70**	10	9	9	9
	**75**	9	9	8	8
	**80**	4	4	3	3
**Strategy III**	**65**	10	10	9	9
	**70**	9	9	8	8
	**75**	8	8	8	7
	**80**	6	5	5	5

1. All three strategies require almost similar amounts of time for process execution and the time is observed to scale as per the number of iterations. In all three strategies, the number of rules appears to reach a plateau after approximately 200–500 iterations depending on the number of samples in the dataset. Overall, results seem to follow a logical pattern and clearly suggest that smaller datasets require higher number of iterations to arrive at a robust set of predictions and vice versa.
2. Strategy III is observed to results in fewer numbers of rules as compared to the other two strategies. Being relatively more stringent in its approach, it is reasonable to adopt strategy III for datasets with higher number of samples. In datasets with very few samples, it is reasonable to expect that this strategy may not generate any association rules. For example, in the prebiotics dataset, the number of rules generated was null (Table 2).

## Discussion

The last decade has witnessed the development of several specialised tools/algorithms catering to various stages of microbiome data analysis viz. host-sequence decontamination [11–13], contig assembly [14–16], taxonomic binning [17–26], functional characterization [27–30], and comparative analysis of microbial communities [31–33]. Beyond elucidating (and comparing) microbial diversity in taxonomic and functional terms, it is important to obtain insights about intra-community microbial interaction patterns and understand the dynamics of these interaction patterns as a function of external environmental changes. The objective of tools/approaches employed for studying microbial community dynamics is to find and characterize microbes (or groups of microbes) that show statistically significant co-presence/co-exclusion patterns. Such patterns find utility in indicating/interpreting (a) synergistic/antagonistic relationships (between various microbes) at a physiological/functional level, and (b) important higher-order community properties such as niche overlap, niche preference, mutualism, competition, amensalism, commensalism etc.

**Fig 6:**
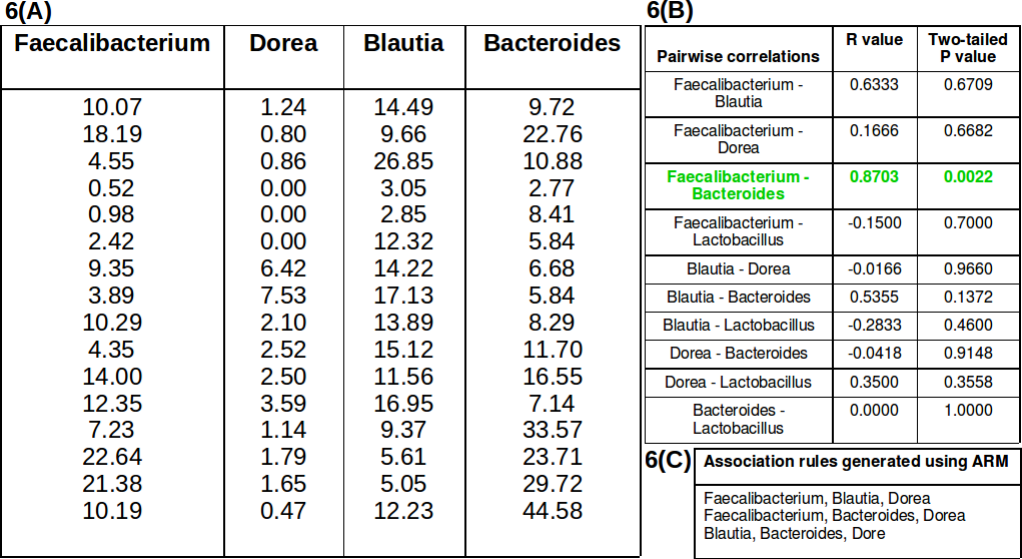
Comparison of results generated using correlation approach and the Apriori approach. A comparison of results generated using (i) correlation approach and (ii) the Apriori approach. The abundance values indicated in part A represent the actual abundances of 4 genera in various samples constituting the prebiotic datasets [2]. Table shown in Part B indicates Spearman correlation values computed between various taxa pairs. The taxon pair that generated a significant correlation is indicated in green font. Part C depicts association rules generated using the Apriori approach.

In silico identification/characterization of association patterns typically involves finding 'statistically significant' co-occurrence/ co-exclusion patterns from a given dataset [34–36]. From the perspective of a biologist, all 'statistically significant' recurrent patterns (i.e. identified microbial associations) may not be 'interesting' from a biological standpoint. On the other hand, several biologically relevant microbial associations may be lost due to over-emphasis on evaluating the statistical significance of a mined association pattern. For instance, consider the abundance profiles of the 4 genera depicted in Fig 6. The abundance values indicated in part A of Fig 6 represent the actual abundances of these 4 genera in various samples constituting the prebiotic datasets [2]. The abundances of genera Faecalibacterium and Blautia (Fig 6A) indicate an absence of a statistically significant correlation (either Pearson or Spearman at a p-value < 0.05) (Fig 6B). However, employing the Apriori rule mining approach on the abundance data (depicted in Fig 6C) results in generation of 'rules' that indicate an associative relationship between these genera (i.e. Faecalibacterium and Blautia). Considering that genera Faecalibacterium and Blautia are known to share a mutual symbiotic relationship [5, 6], ascertaining inter-microbial relationships only on basis of statistical correlation may result in missing out on a few associations that are biologically relevant. In this context, it is important to note that the ultimate objective of the Apriori rule mining approach (like any other predictive approach) is to provide biologists a set of possible interacting (candidate) taxa, the functions of which can be probed for association with the respective phenotype.

Despite its utility in facilitating life sciences researchers to obtain a systems-level understanding of the structure, function and dynamics of microbial communities, employing the Apriori approach for mining association rules from microbiome data has a few limitations. The process of populating/building a 'candidate item set' (with frequent item sets) involves iterative inclusion/extension of one additional item to a frequent subset followed by 'support-count' validation. The iteration continues until no further successful extensions to the frequent subset are found valid from a support count perspective. This procedure necessitates scanning through the same data multiple times, thereby rendering the process computationally inefficient. However, it is important to note that the time taken for generating the candidate item set is not a direct function of data size. The actual number of associations present in the data determines the execution time of the program. Values summarized in Table 1 highlights the latter assertion. As evident from this table, in spite of having 2 times more samples as compared to prebiotic datasets, the time required for processing the HMP datasets (with relatively lesser no. of rules) is 5 times lower that than required for prebiotic datasets.

**Fig 7:**
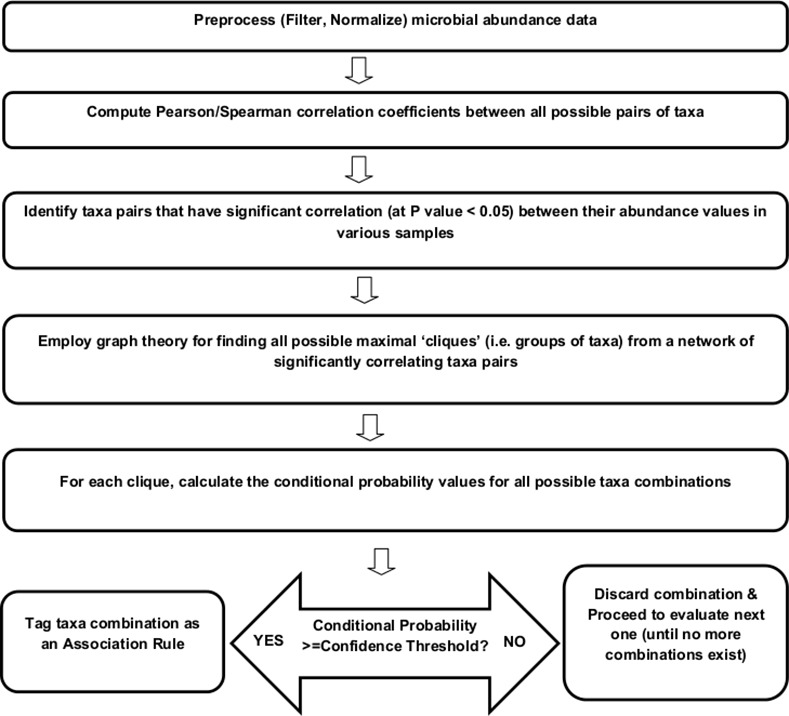
Steps followed in the correlation-based (alternative) rule mining approach. A graphical representation of various steps followed in the correlation-based (alternative) rule mining approach.

As described above, identifying groups of frequently co-occurring features (i.e. frequent item set) constituting the candidate item set is challenging from an implementation perspective. In order to address the challenge associated with this computationally expensive step, an alternative strategy (graphically depicted in Fig 7) for finding association rules was attempted. The alternative strategy involved the following three steps -

Step 1: Identification of significant correlating feature pairs
Step 2: Use of graph theory for finding all possible independent 'cliques' (i.e. groups of features) from such a network of significantly correlating feature pairs
Step 3: Reporting a clique as an 'association rule' if all possible combinations of features in that clique satisfy the 'confidence value' threshold (i.e. conditional probability threshold)

The above strategy was based on the assumption that an ideal candidate set should contain 'only' those features that have a statistically significant correlation (Pearson or Spearman) between their abundance profiles. From a computational perspective, steps 1 and 2 (indicated above) are relatively inexpensive as compared to the process of generating a candidate item set. Step 3 is common to both processes.

Although the alternative strategy showed improved performance in terms of computational efficiency, results summarized in Table 1 indicate poor performance as compared to the association rule mining approach. Several rules identified by the latter approach were observed to be missed by the alternative strategy. In summary, it may again be inferred that following a procedure that initially evaluates the statistical significance of the abundance patterns observed between feature pairs has a high likely-hood of missing several biologically relevant microbial associations that can be identified using association rule mining approaches.

## Conclusions

This study explores and demonstrates the applicability of the 'Apriori algorithm' for deriving 'association rules' from the taxonomic abundance profiles of various samples constituting a given microbiome dataset. The derived rules indicate the pattern of interactions between 'subsets/subgroups' of microbes/taxa constituting these samples. Various customisations performed for adapting the Apriori approach for microbiome data have been described. Results of the validation experiments highlight the efficiency of this 'customised' Apriori approach in deciphering biologically relevant association rules.

## Supporting Information

S1 File Datasets used for evaluating the customized version of Apriori algorithm. A zip archive containing microbial abundance tables which were employed for deciphering association rules using the customised version of the Apriori algorithm.
(ZIP)
